# The Use of Etoposide, Ara-Cytarabine, and Melphalan (EAM) Conditioning Chemotherapy in Autologous Stem Cell Transplantation (ASCT) for a Patient with Relapsed Hodgkin's Lymphoma

**DOI:** 10.1155/2021/9632427

**Published:** 2021-11-03

**Authors:** Eko A. Pangarsa, Ridho M. Naibaho, Vina Yunarvika, Budi Setiawan, Damai Santosa, Catharina Suharti

**Affiliations:** ^1^Subdivion of Hematology and Medical Oncology, Medical Faculty of Diponegoro University, Dr. Kariadi General Hospital, Semarang, Indonesia; ^2^Trainee in Hematology and Medical Oncology, Medical Faculty of Diponegoro University, Dr. Kariadi General Hospital, Semarang, Indonesia; ^3^Department of Internal Medicine, Parikesit Hospital, Mulawarman School of Medicine, Samarinda, East Kalimantan, Indonesia

## Abstract

Up to 20–40% of patients with Hodgkin's lymphoma will eventually relapse after treatment, among which early relapse confers a poor outcome. With salvage chemotherapy followed by autologous stem cell transplantation (ASCT), the long-term remission rate is 30%. We report our experience of using a modified-BEAM conditioning regimen without BCNU consisting of etoposide, cytarabine, and melphalan (EAM) in a patient with relapsed Hodgkin's lymphoma. Before transplantation, the patient achieved second complete remission (CR2) using brentuximab vedotin and ESHAP (BR-ESHAP) chemotherapy. The ASCT went well without significant complications. This case demonstrated the considerable efficacy of EAM protocol as a conditioning regimen in terms of sufficient ablative capabilities, and the patient showed a successful hematopoietic engraftment. Although durability of the disease-free survival needs further observation, it had nearly 18 months of complete remission and the patient was in good performance status at the time of writing this manuscript.

## 1. Background

BEAM (carmustine (BCNU), etoposide, Ara-cytarabine, and melphalan) is considered as the standard conditioning regimen for autologous stem cell transplantation (ASCT) in malignant lymphoma [1, 2]. Unexpectedly, since 2010, the oncological community faced the issue of shortage of some essential chemotherapy drugs, among which was BCNU, one of the central components of the BEAM protocol. Physicians were, thus, forced to change their standard for those regimens in which a component was no longer available: two common solutions were to replace the missing drug with a substitutive agent or even to skip the unavailable drug, trusting that the modified regimen would yield a noninferior result in terms of efficacy and better toxicity profile [3].

BCNU shortage was also reported in Indonesia. So far, we have always been hindered to perform hematopoietic stem cell transplants by the unavailability of this particular drug in the national formularies [4]. We then employed a BEAM-like myeloablative protocol without the letter “B” consisting of etoposide, Ara-cytarabine, and melphalan (EAM) for a preparative ASCT regimen [5], of which one of the cases will be reported here in a patient with Hodgkin's lymphoma.

## 2. Case Presentation

**Figure 1:**
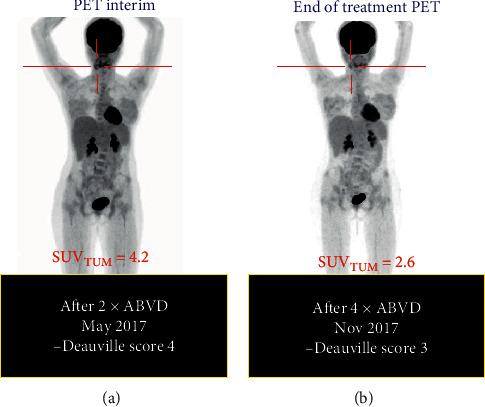
Clinical course of the patient after front-line treatment showing (a) PET-interim partial remission after induction ABVD chemotherapy; (b) PET/CT after completion of 6 cycles reveals complete remission and clinical improvement.

We report a young female patient with bone marrow “only” Hodgkin's lymphoma relapsed within 12 months after front-line ABVD chemotherapy; initial staging was unfavourable, Ann-Arbor II-B with International Prognostic Score (IPS) = 3. A previous pathological report (March 2017) from cervical lymphadenopathy revealed a mixed cellularity classical Hodgkin's lymphoma. Immunohistochemistry showed expression of CD15+, CD30+, CD3+, and high Ki67. The interim evaluation after treatment assessed by an 18-FDG PET scan is shown in Figure 1 and revealed a first complete remission (CR1) upon completion of 6 cycles of ABVD.

**Figure 2:**
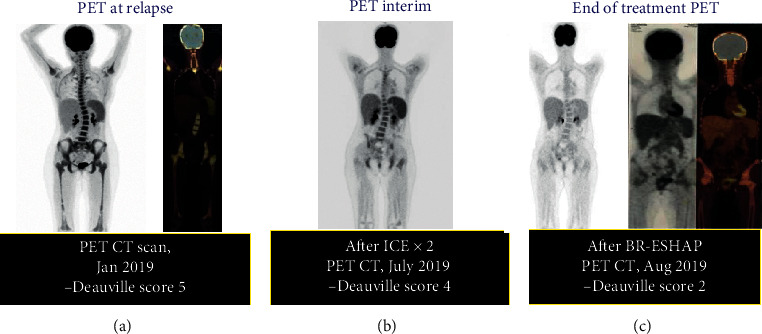
Clinical course of the patient at relapse. (a) First early relapse showing bone marrow progression (Ann-Arbor IVB-E); (b) partial remission after 2 cycles of the first ICE salvage treatment, with faint uptake over the bone marrow; and (c) complete remission after 2 cycles of second salvage using the brentuximab vedotin-ESHAP protocol.

At relapse, she was staged as Ann-Arbor IV-B-E by PET/CT scan (Deauville score 5) and showed bone marrow involvement “only.” As salvage therapy, we treated her with ICE protocol showing partial remission (Interim PET/CT resulted in Deauville score 4) and then we switched to anti-CD30 brentuximab vedotin in combination with ESHAP (BR-ESHAP regimen) for the second salvage. After 2 BR-ESHAP courses, examination by PET/CT demonstrated a negative metabolic activity of the bone marrow (Deauville score 2), see Figure 2.

**Figure 3:**
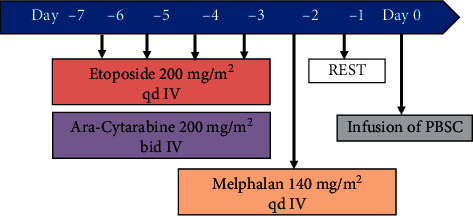
EAM protocol scheme at glance. Preharvested autologous stem cells were infused after 1 full day rest without chemotherapy (day zero). BMT, bone marrow transplantation; PBSC, peripheral blood stem cell.

She agreed to proceed to ASCT with her second complete remission (CR2) status. Peripheral blood stem cell (PBSC) harvest was performed in October 2019 by obtaining a CD34+ of 2.31 × 10^6^ per kg of body weight. Our original uncontrolled rate freezing protocol with only 5% dimethyl sulfoxide (DMSO) by using the Planer 560–16 equipment (Planer products Ltd., UK) was used for stem cell cryopreservation, and the transplant was performed in December 2019. The cryopreserved stem cell was planned to be reinfused to the patient after EAM conditioning at least after 24 hours of free-chemo day. The EAM protocol consisted of intravenous etoposide (200 mg/m^2^ q24 hours) and Ara-cytarabine (200 mg/m^2^ q12 hours) administered for 4 consecutive days (days –6 to –3) followed by melphalan 140 mg/m^2^ given as a single dose at day –2. The protocol and drug sequence administration are illustrated in Figure 3.

**Figure 4:**
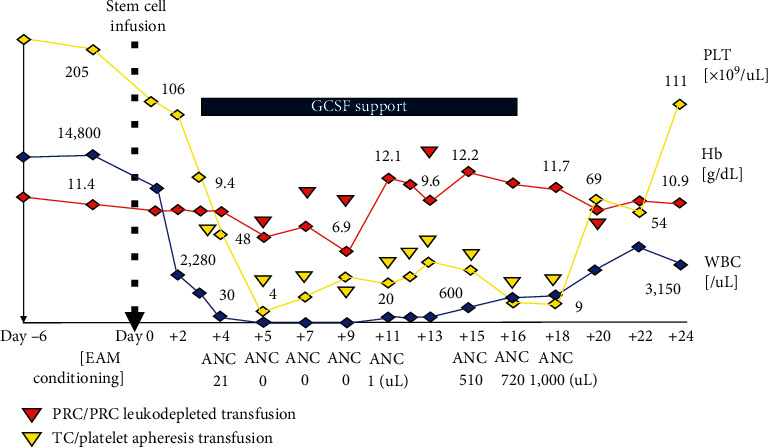
Day-to-day transplant evaluation from conditioning chemotherapy until engraftment. ANC, absolute neutrophil count; Hb, hemoglobin concentration; Plt, platelet count; PRC, packed red cells; and WBC, white blood cell count.

We summarized the transplant course day-by-day until hematopoietic engraftment, as shown in Figure 4. Following EAM chemotherapy and stem cell infusion, the ablative phase of transplant can be observed at day +2. The absolute neutrophil count (ANC) began to decline to <500/*μ*L from day +4, while platelet count kept going down to <20,000/*μ*L and reached its lowest point from day +5. During transplantation, the patient is treated in a positive pressure isolation room and all supportive measures were adopted per local, including the administration of granulocyte colony-stimulating factor (G-CSF) and infectious disease prophylaxis regimen. The posttransplantation period was uneventful though multiple packed red cells and platelet units were required to maintain hemoglobin concentration around 8–10 g/dL and platelet count above 10,000/*μ*L during this critical phase. No other significant nonhematological toxicities except nausea (grade 2), mucositis (grade 2), and diarrhea (grade 1) were reported. Neutrophil engraftment occurred at day +15, and stable platelet count above 20,000/*μ*L was noted from day +20. With a follow-up of 18 months, the patient is in good health with complete and durable hematopoietic resolution with brentuximab vedotin was continued as the consolidation treatment after ASCT.

## 3. Discussion

Hodgkin's lymphoma accounts for approximately 10% of newly diagnosed malignant lymphoma [6]. Up to 20–40% of these patients will eventually relapse after treatment, among which early relapse confers a poor outcome [1, 7]. For patients who exhibited chemosensitivity, salvage treatment and then high-dose (conditioning) chemotherapy followed by ASCT is the current standard treatment [1, 2, 6]. We followed the methods of Wu et al. [8] utilizing brentuximab vedotin therapy to achieve clinical remission. Related to the conditioning regimen, the two randomized controlled phase III clinical studies with BEAM, mini-BEAM, Dexa-BEAM, or high-dose BEAM found that compared with conventional chemotherapy, salvage high-dose chemotherapy combined with ASCT can significantly increase the disease-free survival (DFS) of relapsed/refractory Hodgkin's lymphoma patients [9, 10].

There are several other commonly used regimens as conditioning chemotherapy for malignant lymphoma, namely, high-dose ICE (h-ICE; ifosfamide, carboplatin, and etoposide) [11], CMV (cyclophosphamide, melphalan, and etoposide) [12], CLV (cytarabine, lomustine and etoposide) [13], CBV (cyclophosphamide, BCNU, and etoposide), combination regimens including total body irradiation (TBI) [7, 12], rituximab or iodine-131-tositumomab combined with BEAM [14], and so forth. However, head-to-head comparisons of different conditioning regimens in relapsed/refractory lymphoma patients before ASCT are sparse.

Generally speaking, the BEAM regimen is currently recognized as the best conditioning regimen for Hodgkin's lymphoma [15, 16]. The overall survival (OS) rate of the BEAM pretreatment regimen for Hodgkin's lymphoma patients is the highest with a 3-year progression-free survival (PFS) of 62% and a 3-year OS of 79% [17]. The BEAM regimen has also been compared to three-drug regimen such as CEB (carboplatin, etoposide, and bleomycin) or CLV (cytarabine, lomustine, and etoposide) regimen with better OS in favor of BEAM [13, 18].

Many countries have also experienced temporary shortage of BCNU; the question is that could we simply omit the BCNU. A number of retrospective studies have tried to address the issue. Unfortunately, EAM conditioning in 12 Hodgkin's lymphoma and 11 non-Hodgkin's lymphoma patients by Loke et al. [19] gave disappointing results (mOS 29 months compared to 77 months for the matched BEAM group). A more recent study by Bekadja et al. [20] demonstrated comparable results in efficacy and toxicity with EAM, but the Ara-C total dose was 8 gr/m^2^. Other groups studied the replacement of BCNU in the BEAM regimen by lomustine or thiotepa and stated that although the BEAM group showed a lower rate of transplant-related mortality at day 100, the difference did not reach statistical significance and the 3 regimens had similar results in terms of toxicity and efficacy [21].

This case describes our attempt at modifying the conditioning regimen for relapsed Hodgkin's lymphoma. In the absence of the BCNU component of the BEAM (BCNU, etoposide, cytarabine, and melphalan) [15, 22] or BEAC (BCNU, etoposide, Ara-cytarabine, and cyclophosphamide) [14], the second most important consideration was drug availability while still maintaining effectiveness. The regimen should facilitate a successful transplant, create enough space for preharvested stem cell to regrowth, repopulate, and restore hematopoietic reconstitution or engraftment.

There are several adverse prognostic factors identified on patients with relapsed Hodgkin's lymphoma, among which others are short duration of CR after initial treatment [6]. The five-year-survival rates for early-relapse patients in the German Hodgkin Study Group (GHSG) cohort were below 50% [23]. In this patient, short remission duration (<12 months) was also reported along with presence of B symptoms, extranodal disease, and stage III/IV as predictors for another relapse post-ASCT [24]. A possible explanation for the favorable result of ASCT in the AETHERA study might be due to exclusion of poor-risked patients with refractory disease, good performance status, young age, sufficient stem cell harvest, and no life-threatening toxicity on salvage treatment. According to these clinical pre-ASCT risk factors, we planned to continue brentuximab vedotin up to a total of 16 cycles for better 5-year PFS based on the AETHERA study [25].

In this case report, we ablated the BCNU from the original BEAM regimen without altering either the dosage or drug sequence of the rest component, identical to Loke et al.'s protocol [19]. The details of our protocol can be found elsewhere [5]. Engraftment can be observed in day +15 and day +20 for neutrophils and platelets, respectively. Overall, the EAM scheme was well tolerated. As expected, nausea, mucositis, and diarrhea were the most prominent nonhematologic toxicity and fortunately manageable. These complications occur in frequent occasion with high-intensity regimens [13–23]. Considering the effectiveness of this three-drug regimen in longer-term outcome for PFS or even OS cannot be simply answered from this single case description. But, as depicted in Figure 4, the patient tolerated the expected ablative phase following the EAM regimen, and the cryopreserved stem cells successfully reconstituted hematopoietic engraftment without significant complications. We should admit that the utilization of brentuximab vedotin as the maintenance agent has made a significant survival advantage after ASCT as she is still in good condition until today.

## 4. Conclusions

We report a case of early-relapsed Hodgkin's lymphoma that underwent ASCT following CR2. The main message of this report is that the EAM protocol has the potential to be successful and effectively used in countries with limited access to BCNU. EAM was sufficient in terms of ablative capability to produce space for autograft to restore the hematopoiesis following ASCT.^4^In the meantime, ASCT has not been widely carried out in Indonesia for several reasons; thus, this publication is needed at least to hand in a simpler and feasible conditioning protocol. Further noninferiority trial and prospective study could be specifically designed to provide both a more powerful evidence and survival outcome on the use of EAM in broader clinical practice.

## Data Availability

The data supporting the findings of this report are available from the corresponding author upon reasonable request.

## Abbreviations

ABVD: Adriamycin, bleomycin, vinblastin, and dacarbazine
ANC: Absolute neutrophil count
ASCT: Autologous stem cell transplantation
BCNU: Bis-chloroethyl nitrosurea
BEAC: BCNU (carmustine, etoposide, cytarabine, and cyclophosphamide)
BEAM: BCNU (carmustine, etoposide, cytarabine, and melphalan)
CEB: Cyclophosphamide, etoposide, and BCNU (carmustine)
CBV: Cyclophosphamide, BCNU (carmustine), and etoposide
CLV: Cyclophosphamide, lomustine, and etoposide
CMV: Cyclophosphamide, melphalan, and etoposide
CR: Complete remission
DFS: Disease-free survival
DMSO: Dimethyl sulfoxide
EAM: Etoposide, Ara-cytarabine, and melphalan
G-CSF: Granulocyte colony-stimulating factor
GHSG: German Hodgkin study group
ICE: Ifosfamide, carboplatin, and etoposide
IPS: International Prognostic Score
ESHAP: Etoposide, methylprednisolone, cytarabine, and cisplatin
PBSC: Peripheral blood stem cell
18-FDG PET: Positron emission tomography 18-fluorodeoxyglucose positron emission tomography scan
PFS: Progression-free survival
TBI: Total body irradiation
OS: Overall survival.
